# Iatrogenic acute coronary occlusion during epicardial ventricular tachycardia ablation: when intravascular imaging guides the management—a case report

**DOI:** 10.1093/ehjcr/ytaf358

**Published:** 2025-07-28

**Authors:** Filippo Russo, Ciro Vella, Filippo Maria Cauti, Vittorio Romano, Marco Gamardella, Marco Bruno Ancona, Alaide Chieffo, Matteo Montorfano

**Affiliations:** Interventional Cardiology Unit, IRCCS San Raffaele Scientific Institute, Via Olgettina 60, 20132 Milan, Italy; Interventional Cardiology Unit, IRCCS San Raffaele Scientific Institute, Via Olgettina 60, 20132 Milan, Italy; Department of Cardiac Electrophysiology and Arrhythmology, IRCCS San Raffaele Scientific Institute, Via Olgettina 60, 20132 Milan, Italy; Interventional Cardiology Unit, IRCCS San Raffaele Scientific Institute, Via Olgettina 60, 20132 Milan, Italy; Interventional Cardiology Unit, IRCCS San Raffaele Scientific Institute, Via Olgettina 60, 20132 Milan, Italy; Interventional Cardiology Unit, IRCCS San Raffaele Scientific Institute, Via Olgettina 60, 20132 Milan, Italy; Interventional Cardiology Unit, IRCCS San Raffaele Scientific Institute, Via Olgettina 60, 20132 Milan, Italy; Vita Salute San Raffaele University, IRCCS San Raffaele Scientific Institute, Via Olgettina 60, 20132 Milan, Italy; Interventional Cardiology Unit, IRCCS San Raffaele Scientific Institute, Via Olgettina 60, 20132 Milan, Italy; Vita Salute San Raffaele University, IRCCS San Raffaele Scientific Institute, Via Olgettina 60, 20132 Milan, Italy

**Keywords:** Myocardial infarction, Ventricular tachycardia ablation, Epicardial ablation, Complication, IVUS, Coronary CT, Case report

## Abstract

**Background:**

Ventricular epicardial mapping and ablations pose a significant risk of multiple complications, such as iatrogenic injuries to the coronary arteries.

**Case summary:**

We report a case of acute coronary occlusion during epicardial ventricular tachycardia ablation, detected by ST-segment elevation during the procedure. The multidisciplinary consultation, combined with the use of multimodality imaging, led to the final diagnosis of extrinsic compression caused by a parietal haematoma. This diagnosis guided the decision for conservative management and allowed the team to avoid stent implantation.

**Discussion:**

This case report highlights how multidisciplinary consultation and intravascular ultrasound guidance could lead to a conservative balloon angioplasty treatment strategy, avoiding stent implantation.

Learning points

- Ablation of epicardial ventricular tachycardia is a challenging procedure, but it is crucial to acknowledge the potential complications that can result in ST-segment elevation, which is not always directly related to direct current damage.
- Utilizing multimodal imaging alongside intravascular ultrasound is crucial for effectively addressing these complications and optimizing patient outcomes.

## Introduction

Epicardial substrate involvement is commonly present in non-ischaemic dilated cardiomyopathy (NIDCM) with an inferolateral myocarditis. When the substrate location has an epicardial to endocardial or rarely a transmural extension, an epicardial access during ventricular tachycardia ablation is mandatory to address the scar.^1^ However, mapping and ablating in the epicardial space carry significant risks, including potential injuries to the coronary arteries related to direct radiofrequency (RF) delivery in proximity to the vessels. Another possible complication is linked to a possible phrenic nerve palsy if direct RF is applied on the nerve course.^1^ Because of these dangers, the approach to epicardial ablation and treatment often necessitates a multidisciplinary evaluation, particularly in the event of complications. Utilizing a multidisciplinary team alongside multimodal imaging can be essential for accurately diagnosing and managing complications that may result in ST-segment elevation.

## Summary figure

**Figure:**
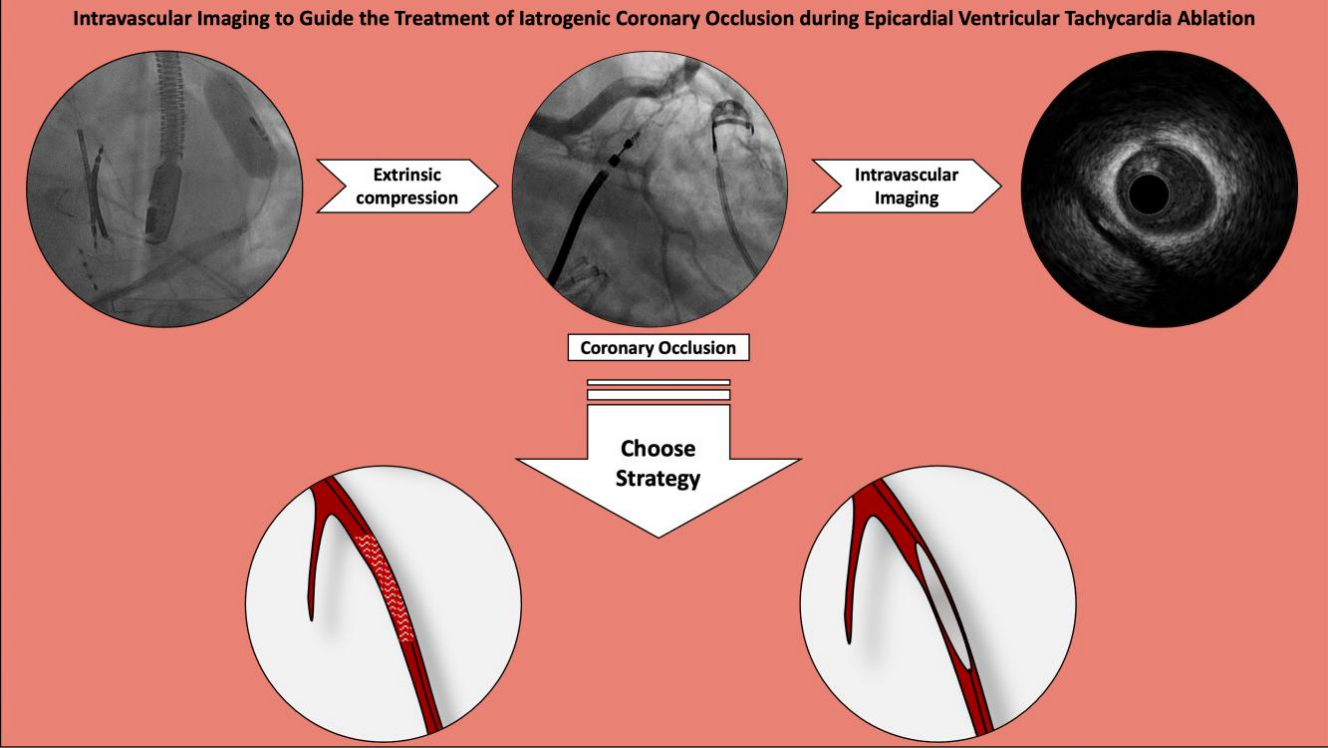


## Manuscript

**Figure 1:**
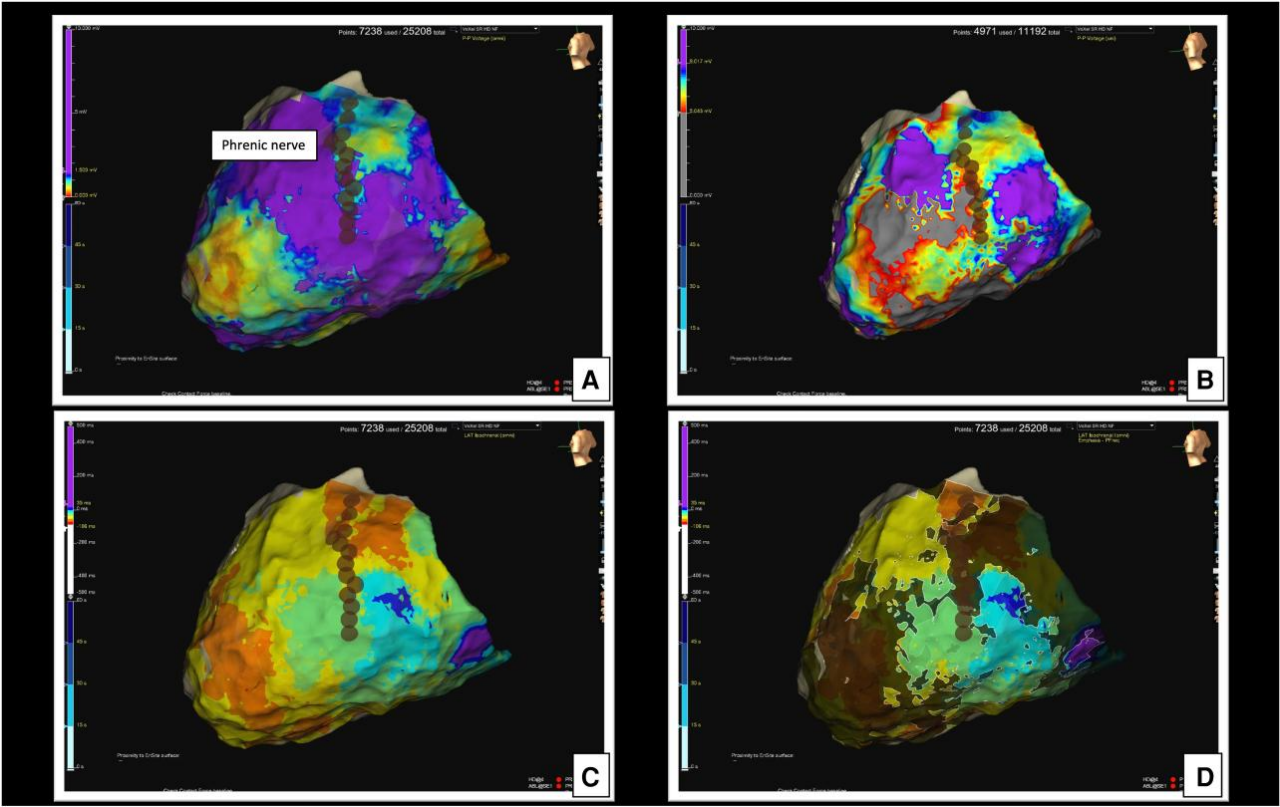
Substrate map acquired during epicardial mapping. (*A*) Omnipolar epicardial map of the LV. (*B*) Unipolar epicardial map of the LV. (*C*) ILAM epicardial map of the LV. (*D*) ILAM epicardial map of the LV with emphasize map.

A 31-year-old male with a history of NIDCM leading to severe left ventricular dysfunction and arrhythmic storms was admitted to our electrophysiology department. In 2022, he experienced an episode of arrhythmic syncope, prompting a cardiovascular magnetic resonance (CMR) that revealed an inferolateral scar with late gadolinium enhancement, primarily affecting the epicardial layer due to a prior episode of myocarditis. Ejection fraction (EF) at the age of presentation was 20%. A prior coronary angiography had ruled out coronary artery disease. As a result, he was implanted with a single-chamber internal cardioverter defibrillator (ICD) for primary prevention. He was treated with beta-blockers, angiotensin receptor–neprilysin inhibitor and sodium–glucose cotransporter-2 inhibitors. One year later, a new ICD lead was placed due to a malfunction of the previous ventricular lead. The patient subsequently experienced multiple episodes of anti-tachycardia pacing and several shocks for fast monomorphic ventricular tachycardia (VT) despite an improvement in EF during the follow-up. A prior 12-lead ECG Holter monitor showed episodes of non-sustained VT that were consistent with an epicardial exit (demonstrated by right bundle branch block morphology with a negative D1 and inferior axis). Upon admission, the antiarrhythmic medications were withdrawn, following our standard protocol. Physical examination was unremarkable. Echocardiogram showed EF 40% with akinesia of the inferolateral wall. Given the CMR findings that demonstrate an epi to endo extension of the scar location, the procedure was planned using an epicardial–endocardial approach. Epicardial puncture was performed under general anaesthesia with a Tuohy needle via a standard inferior approach. A substrate map of the sinus rhythm was obtained using a multipolar HD Grid catheter (*Figure 1A–D*).

**Figure 2:**
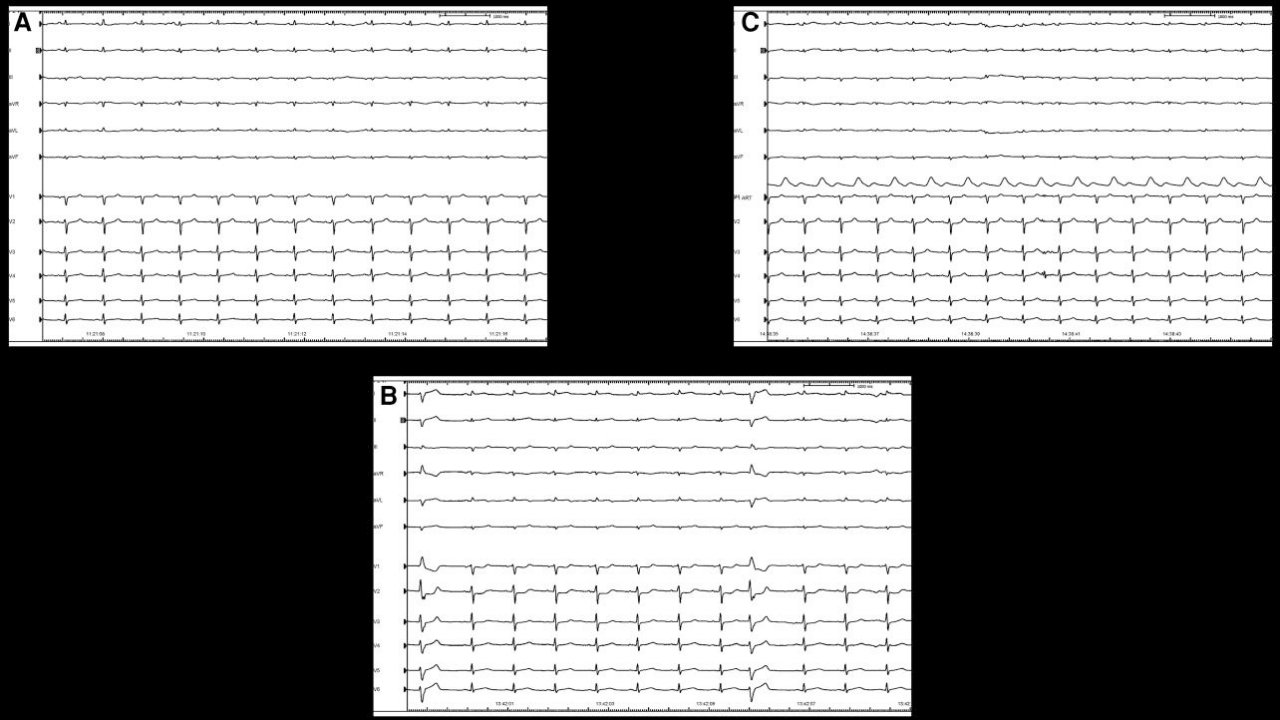
(*A*) Baseline ECG before phrenic nerve displacement. (*B*) ST-segment elevation during the procedure. (*C*) Normalization of the ECG post-angioplasty.

**Figure 3:**
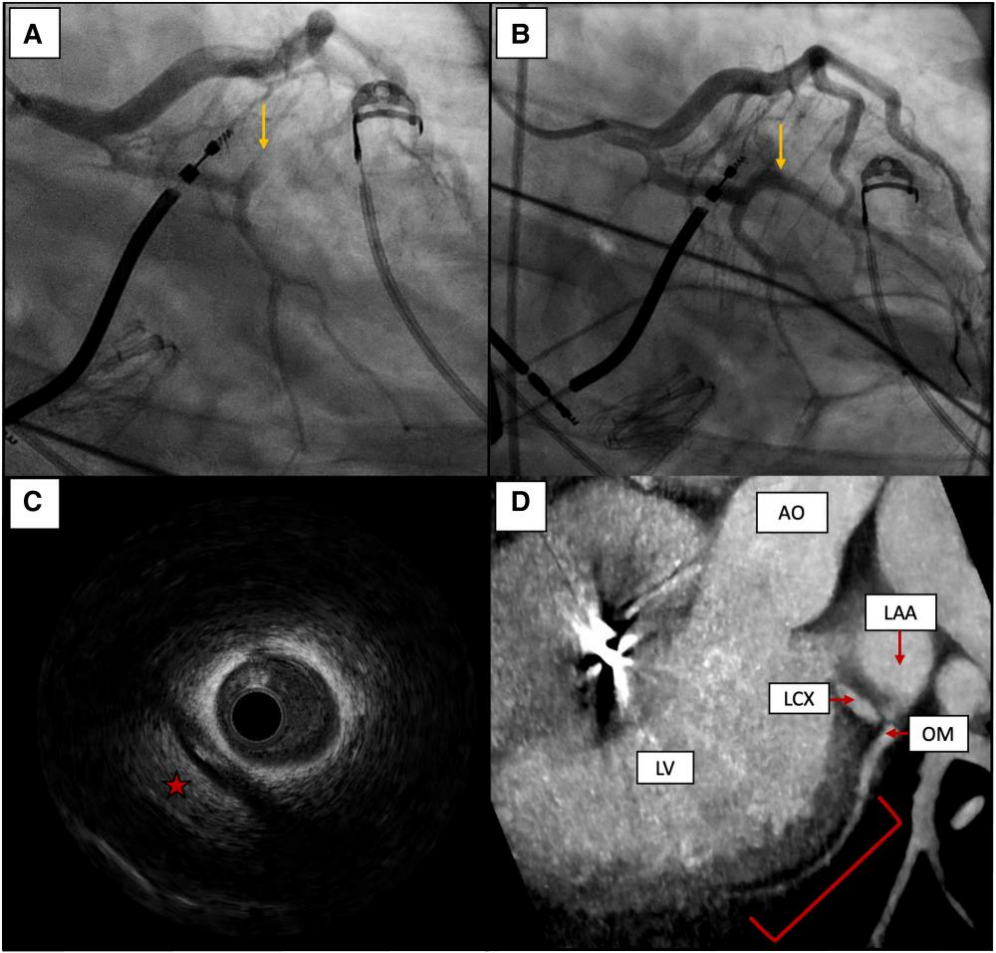
Multimodal imaging of acute coronary occlusion during epicardial ventricular tachycardia ablation. (*A*) Coronary angiography showing occlusion of the OM branch (yellow arrow). (*B*) Residual mild stenosis of the first OM branch after POBA with restored TIMI flow grade 3 (yellow arrow). (*C*) IVUS evidence of iso-hyperechoic extravascular image (red star) with extrinsic compression effect on the vessel. (*D*) CT-scan imaging showing an extended haematoma in the lateral wall of the left ventricle (red parenthesis), compressing the OM.

As every routine during epicardial mapping, especially in the case of a lateral scar, the course of the phrenic nerve was tracked to indicate its position (dots in the map). Our standard approach to functional substrate analysis during sinus rhythm utilizes an isochronal late activation map (ILAM), ventricular electrogram duration map, along with late potentials and peak frequency analysis, as previously described.^2–5^ An attempt to induce ventricular arrhythmias was made even after the infusion of isoproterenol, but no sustained arrhythmias occurred. After a careful analysis of the scar and the functional substrate, an extensive area, part of which was located underneath the course of the phrenic nerve, was targeted. To facilitate this, we obtained double pericardial access and inserted a valvuloplasty balloon to intentionally displace the phrenic nerve in the area of interest (Osypka VACII, 20 × 40). This manoeuvre is crucial to prevent phrenic nerve injury due to radiofrequency ablation and is part of our standard protocol in such cases. Ablation was performed using a Tactiflex Curve D irrigated catheter (Abbott) in dragging mode, with a power setting of 50 W and an irrigation flow rate of 10 mL/min, in an area not adjacent to the phrenic nerve. After eliminating the substrate far away from the phrenic nerve, the balloon was sequentially inflated to further displace it. The RF energy delivered beneath the valvuloplasty balloon was in power control mode (50 W), with isolated RF pulses lasting a maximum of 40 s. The balloon was slightly deflated to allow for repositioning of the RF catheter, and this process was repeated until the desired modification of the substrate was achieved. During these manoeuvres, ST-segment elevation was documented in leads D1 and aVL, with corresponding ST-segment depression observed in leads V1–V2 (*Figure 2A* and *B*). Coronary angiography was performed immediately afterwards and showed no evidence of atherosclerotic disease but revealed acute occlusion of the first obtuse marginal branch (MO), with thrombolysis in myocardial infarction (TIMI) 0 flow (*Figure 3A*; Supplementary material online, *Video S1*). A workhorse coronary guidewire was utilized to cross the lesion, where significant resistance was noticed during navigation through the ‘culprit’ lesion. A 2.0 mm balloon was inflated, achieving vessel recanalization, but residual narrowing persisted at the site of the culprit lesion. To exclude coronary artery spasm as a potential cause of transient ST elevation, intracoronary nitrate was administered without evidence of restoring the normal vessel diameter. Further prolonged inflation with a 2.5 mm balloon resulted in TIMI 3 flow and complete resolution of the ST-segment elevation (*Figures 2C* and *3B*), albeit with mild residual stenosis (Supplementary material online, *Video S2*).

Intravascular ultrasound (IVUS) imaging revealed a peri-vascular haematoma causing external compression of the vessel, without any presence of thrombus, atherosclerotic plaque, or dissection (*Figure 3C*; Supplementary material online, *Video S3*). Considering the IVUS findings, the satisfactory angiographic result, the normalization of the ST-segment, and the young age of the patient, we decided against stent implantation. Next, we performed an endocardial map to confirm the absence of a substrate target for ablation. We conducted programmed ventricular stimulation from the right ventricular apex as well as left ventricular (LV) endo- and epicardial sites to assess for non-inducibility at the end of the procedure. On the second postoperative day, a computer tomography (CT) scan confirmed both the patency of the vessel and the presence of haematoma in the peri-vascular area (*Figure 3D*). The subsequent hospitalization was uneventful, with elevated troponin levels noted primarily due to the radiofrequency ablation. The patient was discharged in good clinical condition on single antiplatelet therapy. No arrhythmias were recorded during the hospital stay. One month later, the single antiplatelet therapy was discontinued without any complications. After 16 months of follow-up, the patient remained free of arrhythmias and showed no coronary abnormalities. No ECG or echocardiogram modifications were noted.

## Discussion

Acute coronary occlusion during epicardial ventricular tachycardia ablation is a rare complication, leading to ST elevation.

While direct heating of the RF is typically assumed to be the cause of coronary damage during epicardial ablation, mechanical extrinsic compression by parietal haematoma, as in this case, may also contribute.

However, in these critical settings, the right management is unclear, and in some cases, reported stent implantation as a possible solution.^6,7^

Our case reports for the first time highlight how IVUS and other intracoronary imaging tools could help to clarify the aetiology. This crucial step can lead to a conservative balloon angioplasty treatment strategy, avoiding stent implantation, when iatrogenic pathophysiology is confirmed, and complications such as plaque complications, thrombus, and vessel dissection are excluded.

Finally, visualization of coronary arteries through coronary angiography or angioCT scan is advised before performing epicardial ventricular arrhythmias ablation in areas with possible proximity to coronary arteries.^8^

We therefore suggest a multidisciplinary management and the introduction of a standard protocol with IVUS guidance in these critical settings to confirm the cause of ST elevation and to guide the strategy of revascularization.

## Supplementary Material

ytaf358_Supplementary_Data

## Data Availability

The data underlying this article are available upon reasonable request.

## Lead author biography

Filippo Russo, MD, is Physician, Specialist in Cardiology. He is Associate Medical Director of the Interventional Cardiology Unit at IRCCS San Raffaele Scientific Institute, Milan, Italy. Dr Russo is expert operator in coronary and structural heart fields and is author of numerous scientific publications in international journals and book chapters in the field of interventional cardiology.
